# Supplementary material for: Different Ecological Niches of Poisonous Aristolochia clematitis in Central and Marginal Distribution Ranges—Another Contribution to a Better Understanding of Balkan Endemic Nephropathy
Source: Plants (Basel). 2023 Aug 22;12(17):3022. doi: 10.3390/plants12173022 (PMC10489678; doi:10.3390/plants12173022)
Supplement: Supplementary file 1 [file plants-12-03022-s001.zip › Figure S1.pdf]

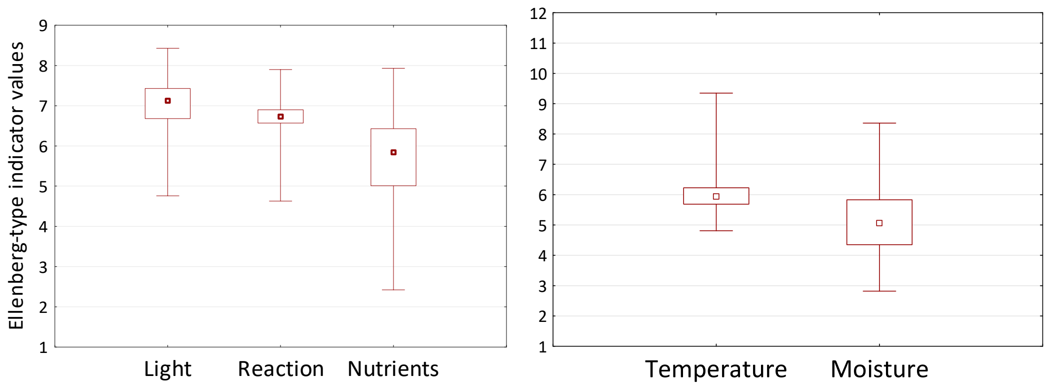

Figure S1. Ellenberg-type indicator values for the whole ecological niche of *Aristolochia clematitis* calculated on a whole dataset (851 vegetation plots). Boxes show the 25-75% quartile range and the median value, whiskers indicate the range of values.
