# Supplementary material for: Different Ecological Niches of Poisonous Aristolochia clematitis in Central and Marginal Distribution Ranges—Another Contribution to a Better Understanding of Balkan Endemic Nephropathy
Source: Plants (Basel). 2023 Aug 22;12(17):3022. doi: 10.3390/plants12173022 (PMC10489678; doi:10.3390/plants12173022)
Supplement: Supplementary file 1 [file plants-12-03022-s001.zip › Figure S2.pdf]

A

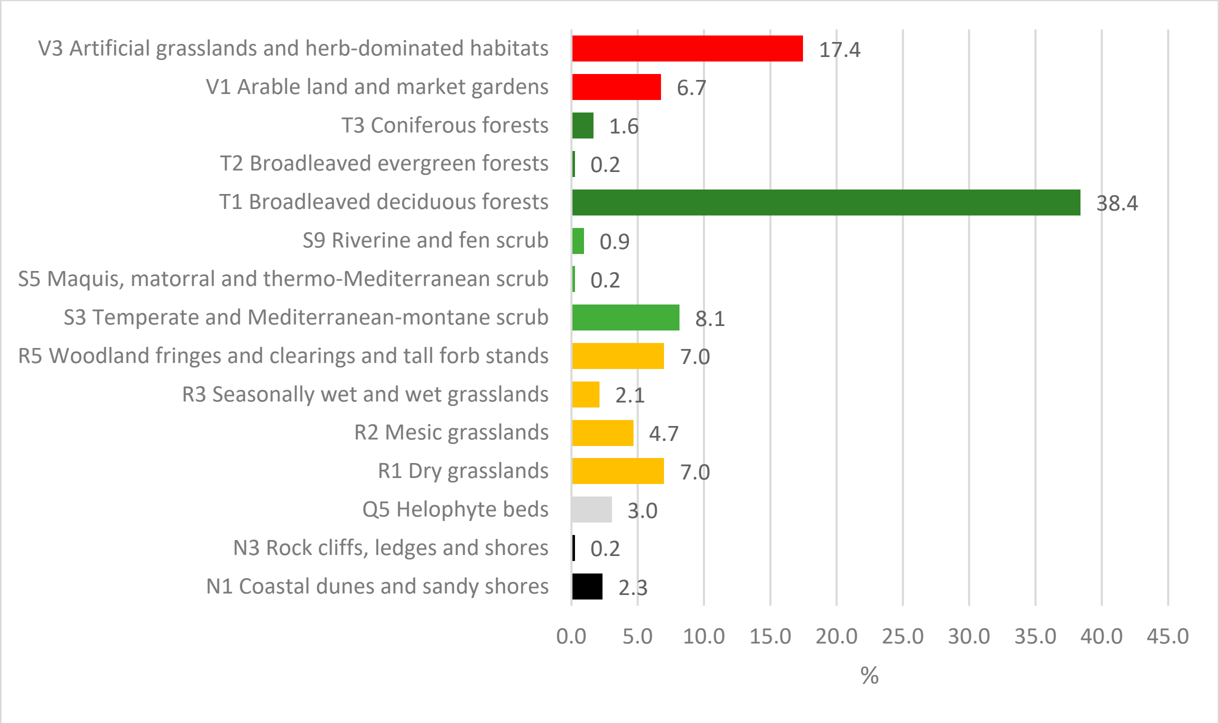
V3 Artificial grasslands and herb-dominated habitats 17.4
V1 Arable land and market gardens 6.7
T3 Coniferous forests 1.6
T2 Broadleaved evergreen forests 0.2
T1 Broadleaved deciduous forests 38.4
S9 Riverine and fen scrub 0.9
S5 Maquis, matorral and thermo-Mediterranean scrub 0.2
S3 Temperate and Mediterranean-montane scrub 8.1
R5 Woodland fringes and clearings and tall forb stands 7.0
R3 Seasonally wet and wet grasslands 2.1
R2 Mesic grasslands 4.7
R1 Dry grasslands 7.0
Q5 Helophyte beds 3.0
N3 Rock cliffs, ledges and shores 0.2
N1 Coastal dunes and sandy shores 2.3
0.0 5.0 10.0 15.0 20.0 25.0 30.0 35.0 40.0 45.0
%

B

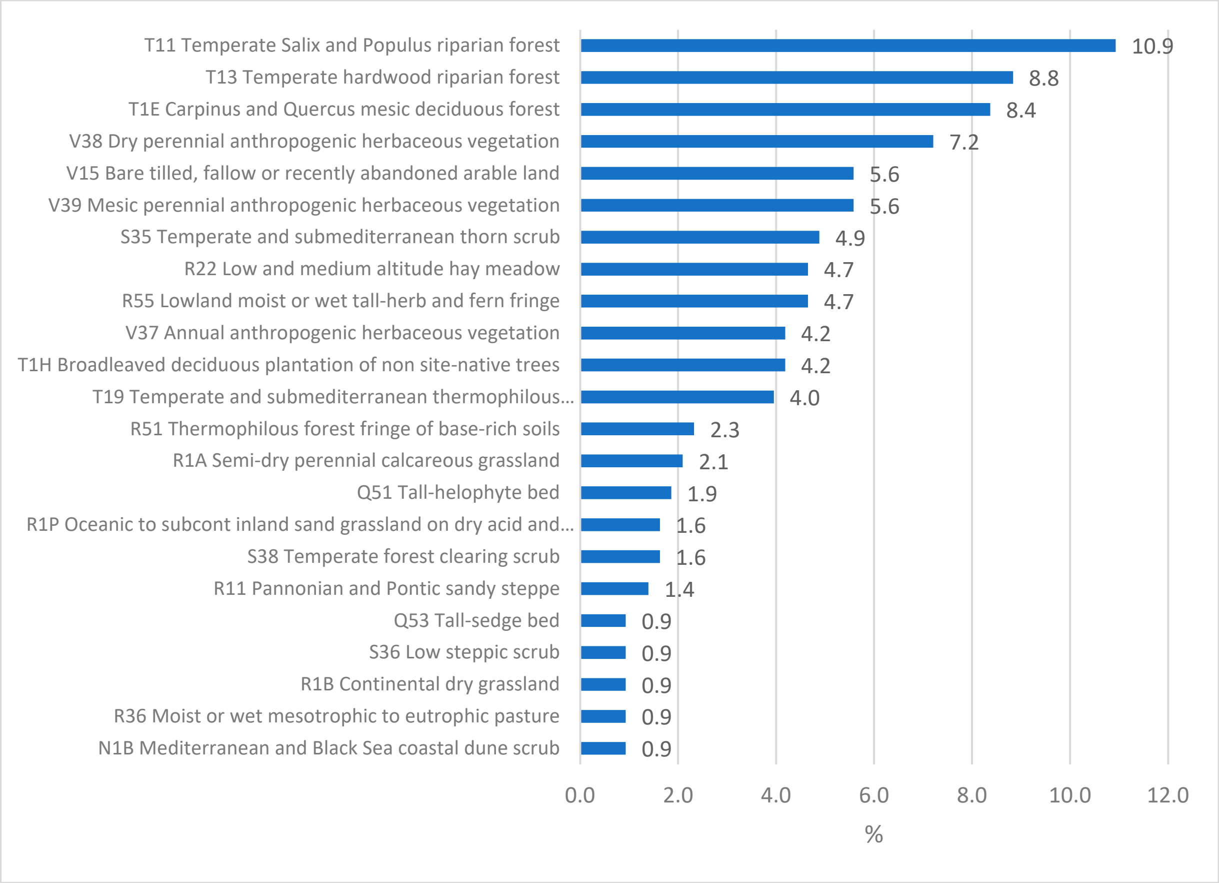
T11 Temperate Salix and Populus riparian forest 10.9
T13 Temperate hardwood riparian forest 8.8
T1E Carpinus and Quercus mesic deciduous forest 8.4
V38 Dry perennial anthropogenic herbaceous vegetation 7.2
V15 Bare tilled, fallow or recently abandoned arable land 5.6
V39 Mesic perennial anthropogenic herbaceous vegetation 5.6
S35 Temperate and submediterranean thorn scrub 4.9
R22 Low and medium altitude hay meadow 4.7
R55 Lowland moist or wet tall-herb and fern fringe 4.7
V37 Annual anthropogenic herbaceous vegetation 4.2
T1H Broadleaved deciduous plantation of non site-native trees 4.2
T19 Temperate and submediterranean thermophilous... 4.0
R51 Thermophilous forest fringe of base-rich soils 2.3
R1A Semi-dry perennial calcareous grassland 2.1
Q51 Tall-helophyte bed 1.9
R1P Oceanic to subcont inland sand grassland on dry acid and... 1.6
S38 Temperate forest clearing scrub 1.6
R11 Pannonian and Pontic sandy steppe 1.4
Q53 Tall-sedge bed 0.9
S36 Low steppic scrub 0.9
R1B Continental dry grassland 0.9
R36 Moist or wet mesotrophic to eutrophic pasture 0.9
N1B Mediterranean and Black Sea coastal dune scrub 0.9
0.0 2.0 4.0 6.0 8.0 10.0 12.0
%

C

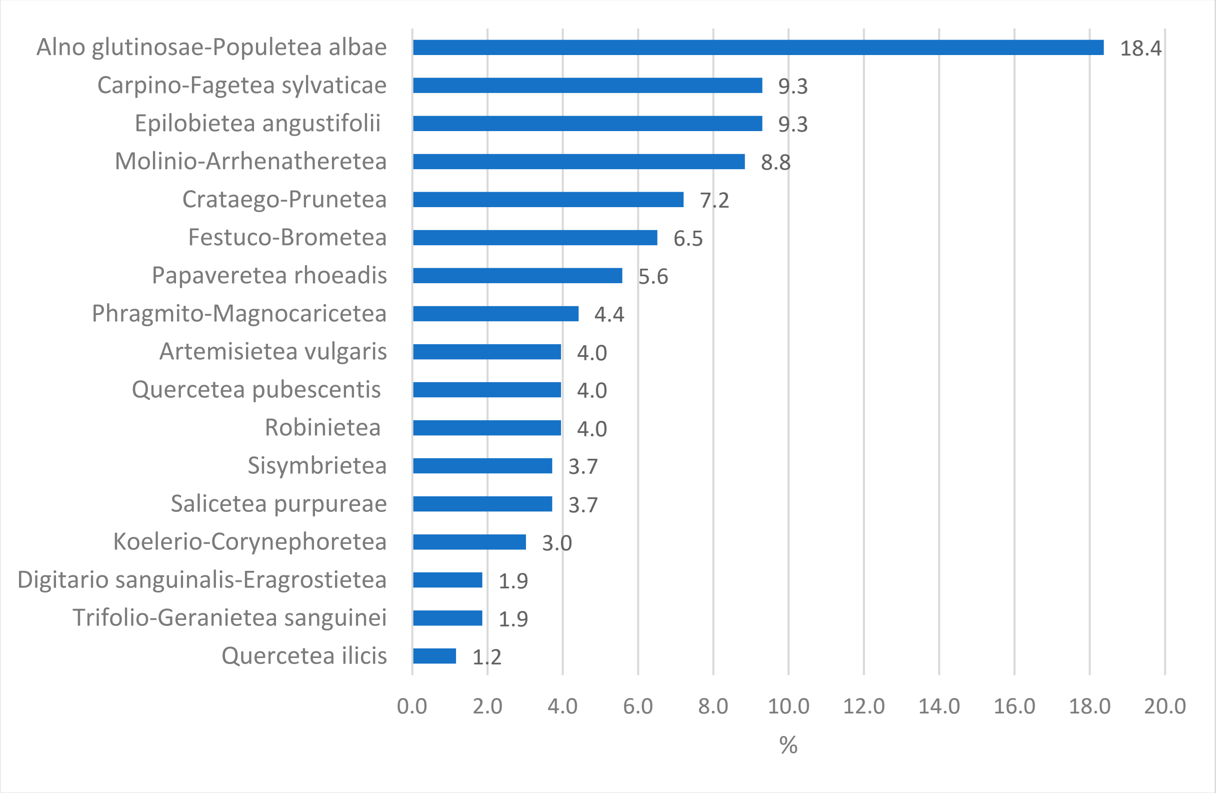

Figure S2. Percentage of EUNIS habitat types and vegetation classes in the whole dataset (851 vegetation plots)

A Percentage of EUNIS habitat types (level 2)

B Percentage of the most frequent EUNIS habitat types (level 3)

C Percentage of the most frequent vegetation classes
