# Supplementary material for: Different Ecological Niches of Poisonous Aristolochia clematitis in Central and Marginal Distribution Ranges—Another Contribution to a Better Understanding of Balkan Endemic Nephropathy
Source: Plants (Basel). 2023 Aug 22;12(17):3022. doi: 10.3390/plants12173022 (PMC10489678; doi:10.3390/plants12173022)
Supplement: Supplementary file 1 [file plants-12-03022-s001.zip › Figure S3.pdf]

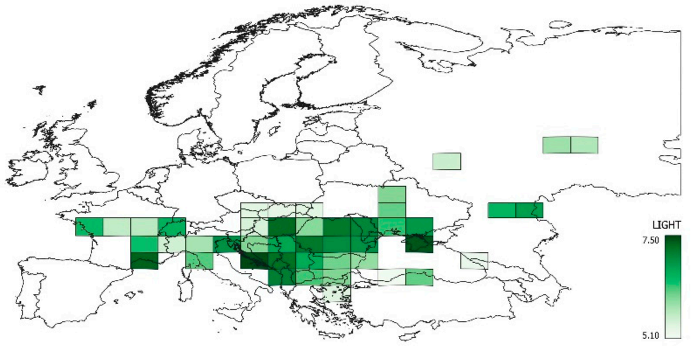

A Ellenberg-type indicator values for Light

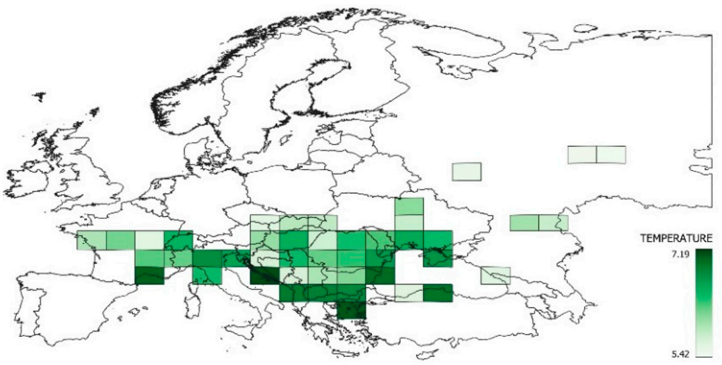

B Ellenberg-type indicator values for Temperature

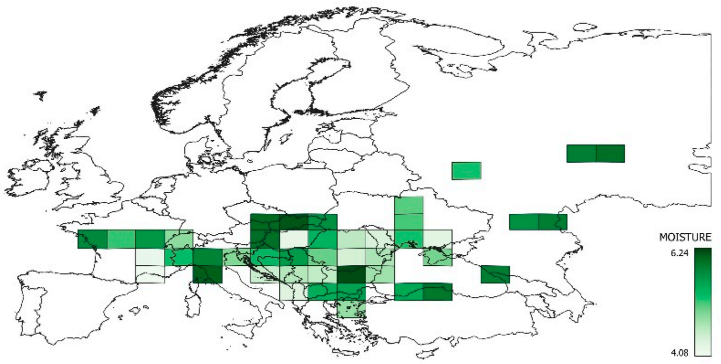

C Ellenberg-type indicator values for Moisture

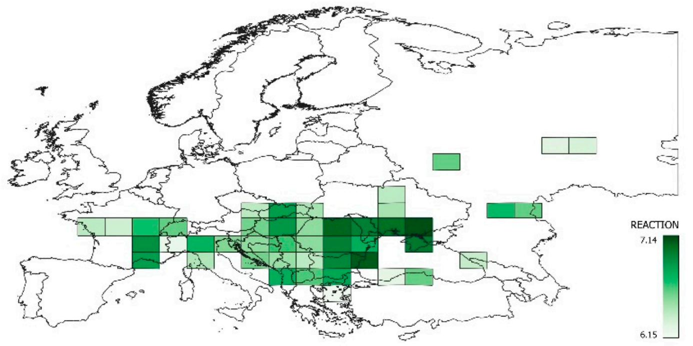

D Ellenberg-type indicator values for Soil Reaction

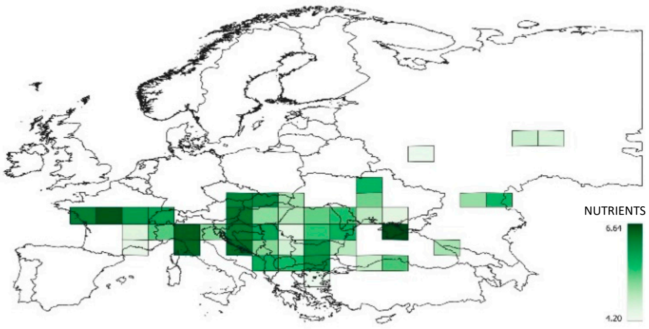

E Ellenberg-type indicator values for Soil Nutrients

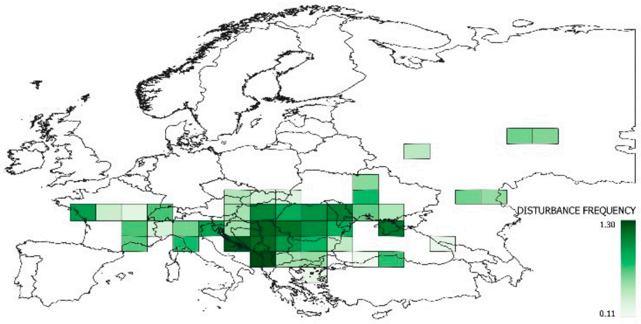

F Indicator values for Disturbance Frequency

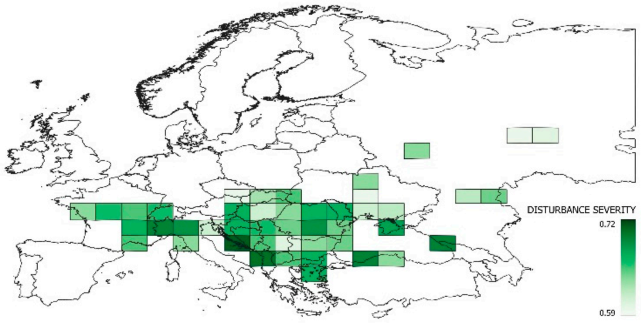

G Indicator values for Disturbance Severity

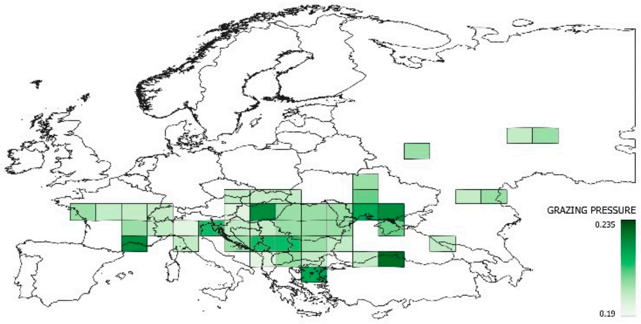

H Indicator values for Grazing Pressure

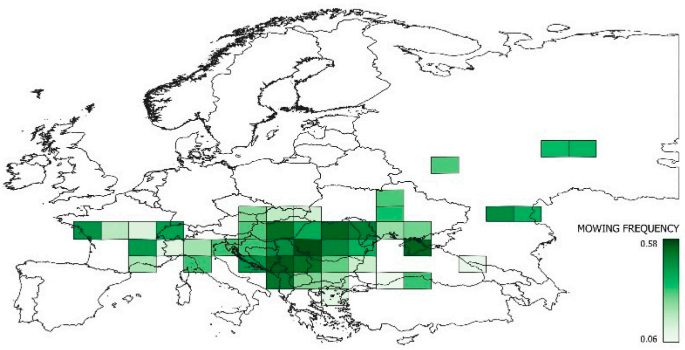

I Indicator values for Mowing Frequency

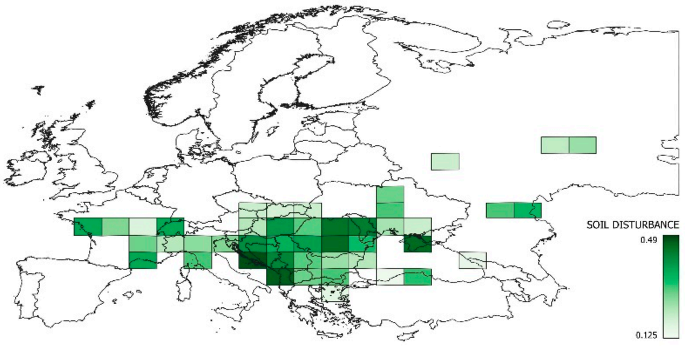

J Indicator values for Soil Disturbance

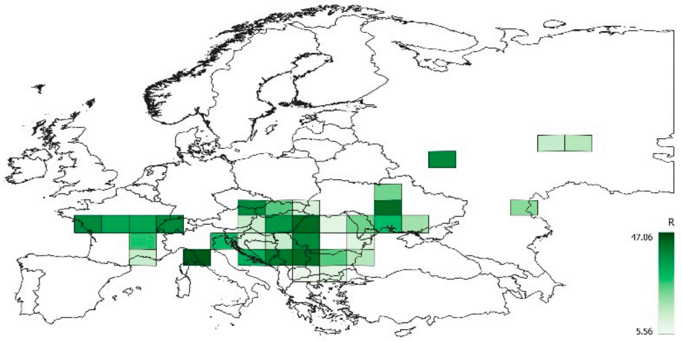

K Percentage of grassland habitatas

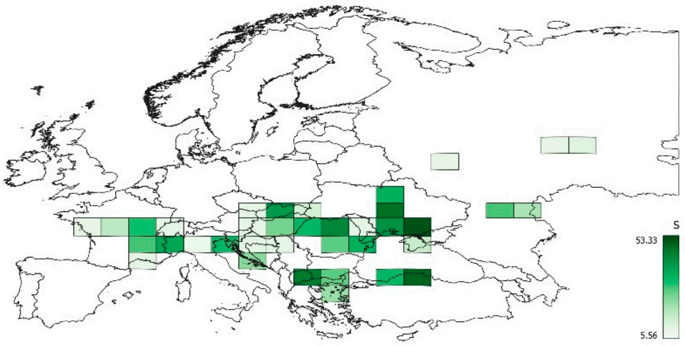

L Percentage of scrub habitatas

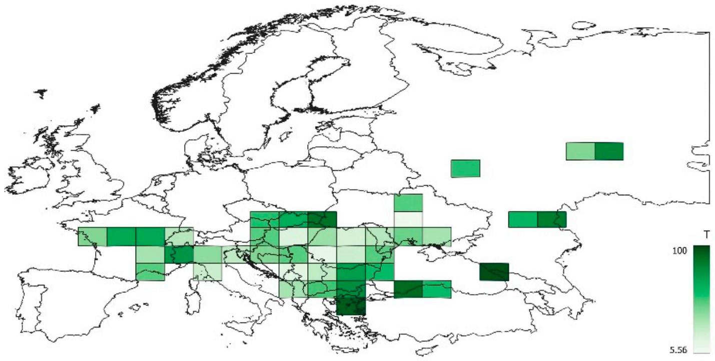

M Percentage of forest habitatas

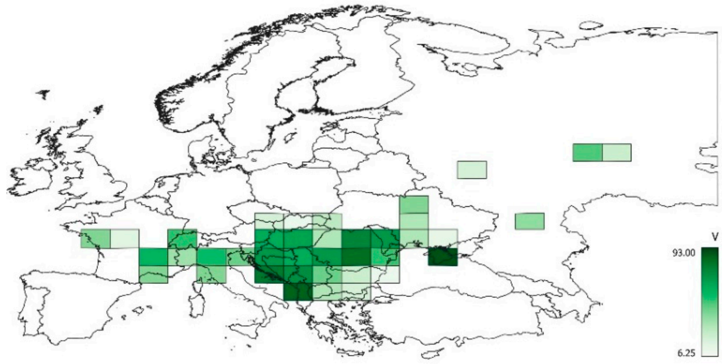

N Percentage of man-made habitats

Figure S3. Medians of environmental variables across MGRS cells
