# Supplementary material for: Different Ecological Niches of Poisonous Aristolochia clematitis in Central and Marginal Distribution Ranges—Another Contribution to a Better Understanding of Balkan Endemic Nephropathy
Source: Plants (Basel). 2023 Aug 22;12(17):3022. doi: 10.3390/plants12173022 (PMC10489678; doi:10.3390/plants12173022)
Supplement: Supplementary file 1 [file plants-12-03022-s001.zip › Figure S4.pdf]

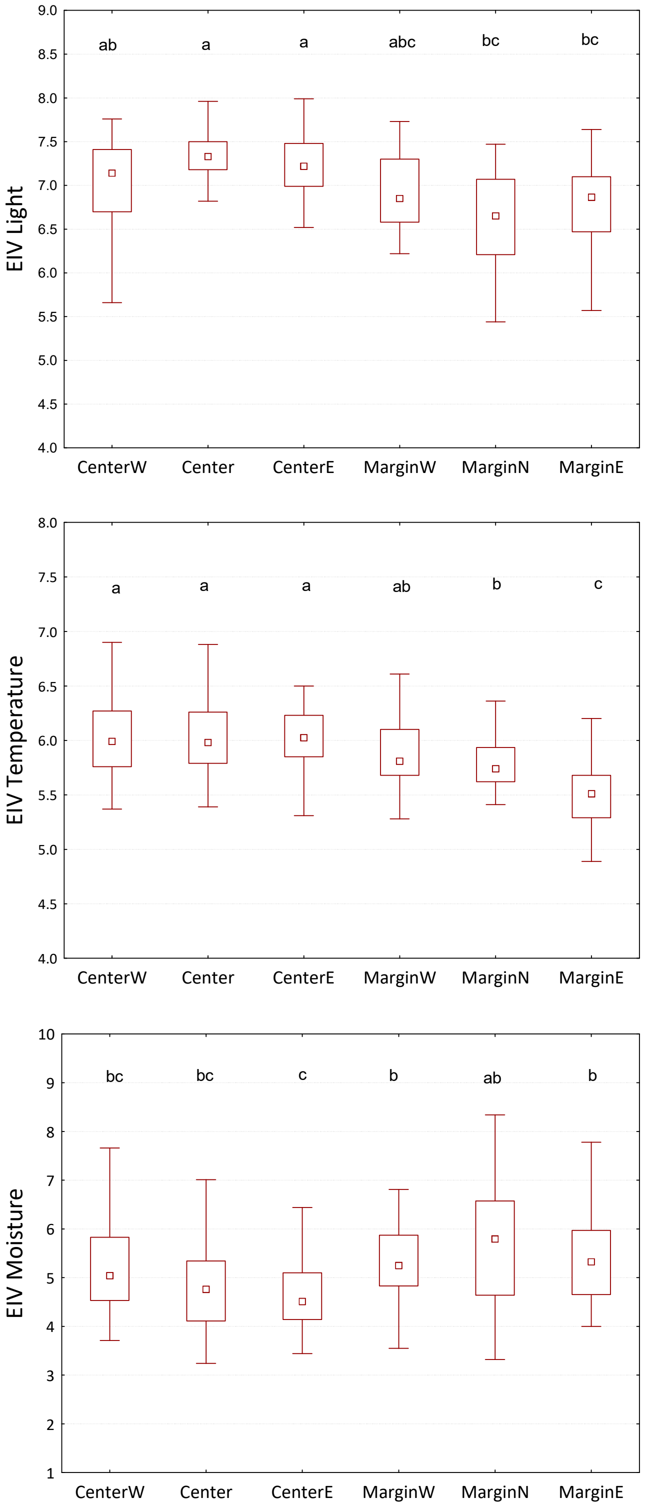
EIV Light
9.0
8.5
8.0
7.5
7.0
6.5
6.0
5.5
5.0
4.5
4.0
ab
a
a
abc
bc
bc
CenterW
Center
CenterE
MarginW
MarginN
MarginE
EIV Temperature
8.0
7.5
7.0
6.5
6.0
5.5
5.0
4.5
4.0
a
a
a
ab
b
c
CenterW
Center
CenterE
MarginW
MarginN
MarginE
EIV Moisture
10
9
8
7
6
5
4
3
2
1
bc
bc
c
b
ab
b
CenterW
Center
CenterE
MarginW
MarginN
MarginE

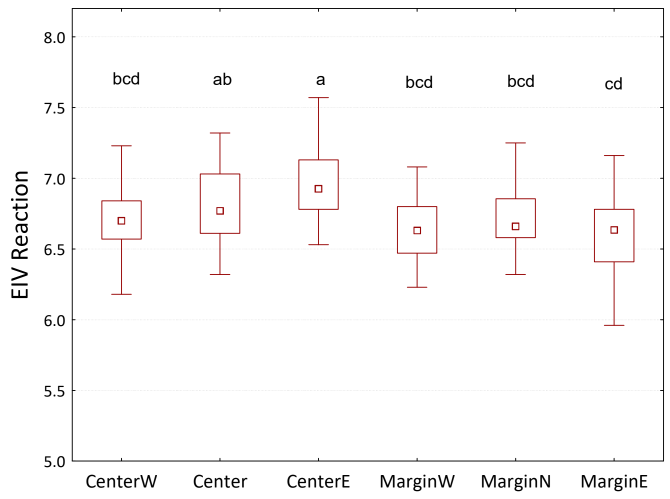
EIV Reaction
8.0
7.5
7.0
6.5
6.0
5.5
5.0
bcd
ab
a
bcd
bcd
cd
CenterW
Center
CenterE
MarginW
MarginN
MarginE

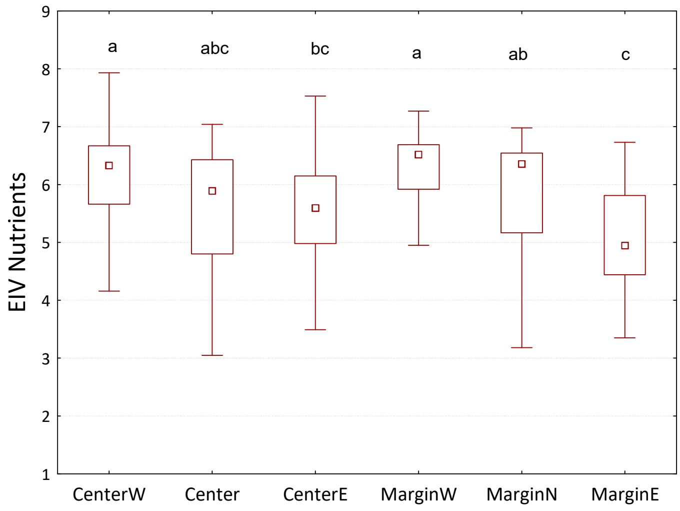
EIV Nutrients
9
8
7
6
5
4
3
2
1
a
abc
bc
a
ab
c
CenterW
Center
CenterE
MarginW
MarginN
MarginE

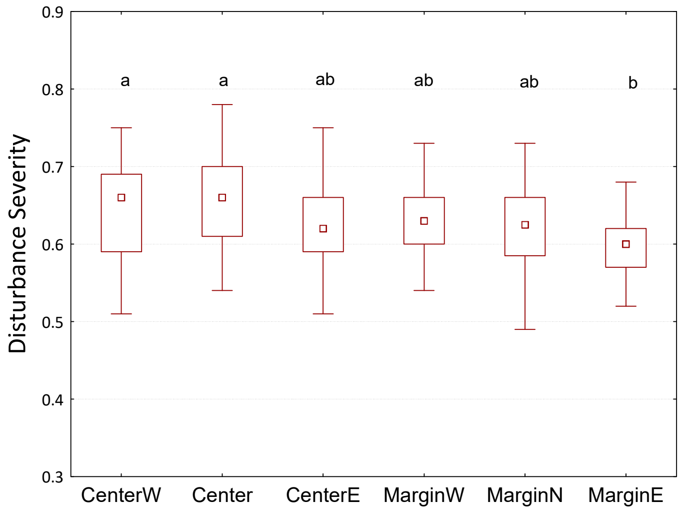
Disturbance Severity
0.9
0.8
0.7
0.6
0.5
0.4
0.3
a
a
ab
ab
ab
b
CenterW
Center
CenterE
MarginW
MarginN
MarginE

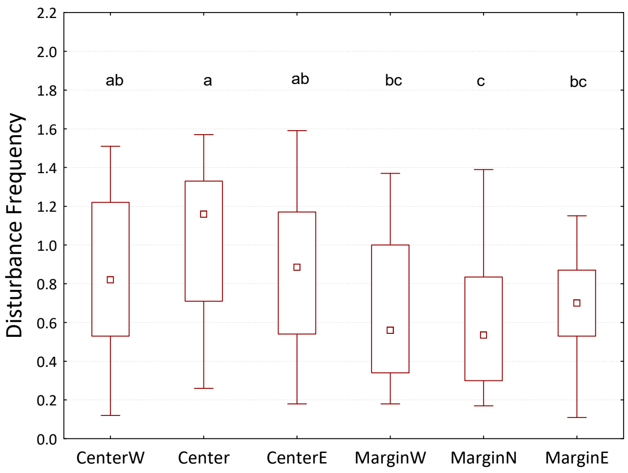
Disturbance Frequency
2.2
2.0
1.8
1.6
1.4
1.2
1.0
0.8
0.6
0.4
0.2
0.0
ab
a
ab
bc
c
bc
CenterW
Center
CenterE
MarginW
MarginN
MarginE

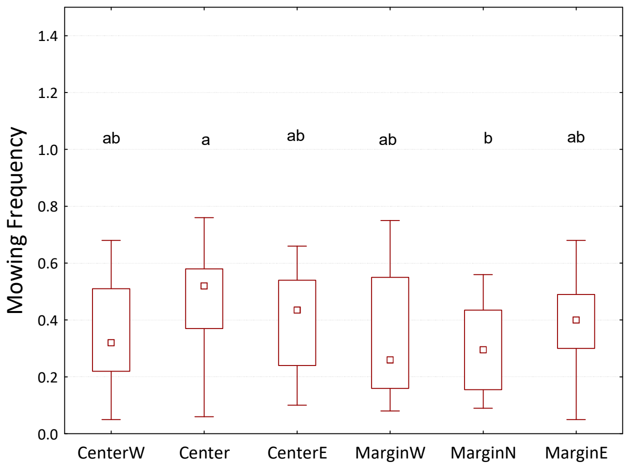
Mowing Frequency
1.4
1.2
1.0
0.8
0.6
0.4
0.2
0.0
ab
a
ab
ab
b
ab
CenterW
Center
CenterE
MarginW
MarginN
MarginE

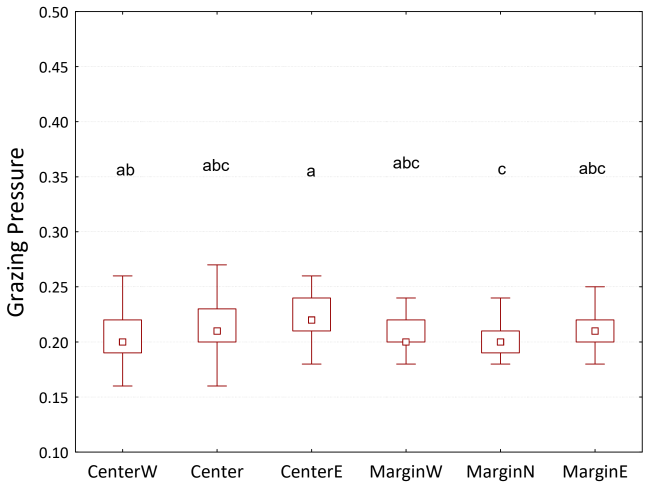
Grazing Pressure
0.50
0.45
0.40
0.35
0.30
0.25
0.20
0.15
0.10
ab
abc
a
abc
c
abc
CenterW
Center
CenterE
MarginW
MarginN
MarginE

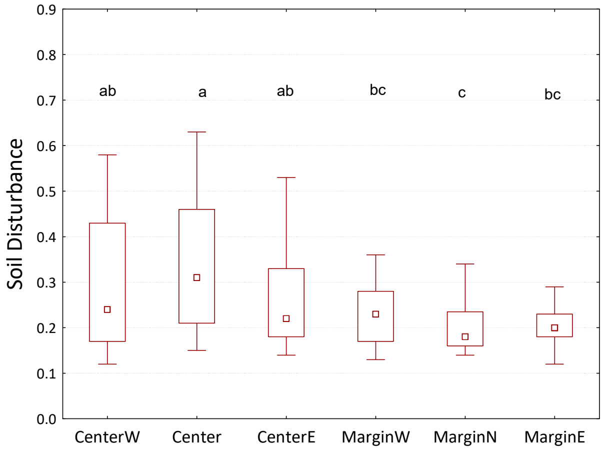

Figure S4. Ellenberg-type and disturbance indicator values of the vegetation plots classified into three marginal and three central regions. Boxes show the 25-75% quartile range and the median value, whiskers indicate the range of values.
