# Supplementary material for: Different Ecological Niches of Poisonous Aristolochia clematitis in Central and Marginal Distribution Ranges—Another Contribution to a Better Understanding of Balkan Endemic Nephropathy
Source: Plants (Basel). 2023 Aug 22;12(17):3022. doi: 10.3390/plants12173022 (PMC10489678; doi:10.3390/plants12173022)
Supplement: Supplementary file 1 [file plants-12-03022-s001.zip › Table S1.pdf]

Table S1. Contributing vegetation-plot databases with Global Index Vegetation-plot Databases (GIVD; www.givd.info) codes, names and number of their plots included in the final stratified and resampled dataset (final number of plots = 851). NA – databases not included in GIVD

GIVD code	Database name	No. of veg. plots	Custodian
EU-FR-003	SOPHY	77	Emmanuel Garbolino
EU-RO-008	Romanian Grassland Database	77	Eszter Ruprecht
EU-00-019	Balkan Vegetation Database	52	Kiril Vassilev
EU-00-028	European Weed Vegetation Database	51	Filip Küzmič
EU-RU-011	Vegetation Database of Tatarstan	39	Vadim Prokhorov
EU-RU-002	Lower Volga Valley Phytosociological Database	38	Valentin Golub
EU-SK-001	Slovak Vegetation Database	34	Milan Valachovič
EU-UA-011	Ukrainian Anthropogenic VDB	34	Tetiana Dziuba
00-TR-003	Non-Forest Vegetation Database of Turkey - NFVDT	30	Behlül Güler
EU-00–030	Eastern European Steppe Database	26	Denys Vynokurov
EU-HR-002	Croatian Vegetation Database	25	Željko Škvorc
EU-HU-003	CoenoDat Hungarian Phytosociological Database	23	János Csiky
EU-RO-007	Romanian Forest Database	23	Adrian Indreica
EU-IT-021	AMS-VegBank - Alma Mater Studiorum - University of Bologna	20	Alessandro Chiarucci
EU-UA-001	Ukrainian Grassland Database	18	Anna Kuzemko
EU-00-026	CircumMed Forest database	17	Gianmaria Bonari
EU-IT-011	Vegetation Plot Database - Sapienza University of Rome	17	Emiliano Agrillo
EU-00-013	Balkan Dry Grasslands Database	16	Kiril Vassilev
EU-RU-014	Temperate Forests of European Russia	16	Larisa Khanina
EU-IT-001	VegItaly	15	Roberto Venanzoni
EU-00-027	European Boreal Forest Vegetation Database	14	Anni Kanerva Jašková
EU-GR-005	Hellenic Natura 2000 Vegetation Database (HelNatVeg)	14	Panayotis Dimopoulos
EU-00-031	Masaryk University's Gap-Filling Database of European Vegetation	13	Milan Chytrý
EU-UA-006	Vegetation Database of Ukraine and Adjacent Parts of Russia	12	Viktor Onyshchenko
EU-FR-004	VEGFRANCE	12	Jan-Bernard Bouzillé
EU-UA-005	Halophytic and coastal vegetation database of Ukraine	11	Tetiana Dziuba
EU-CZ-001	Czech National Phytosociological Database	9	Milan Chytrý
EU-UA-010	Ukraine Psammophytic VDB	9	Tetiana Dziuba
EU-FR-006	SIMETHIS-Flore-CBNMed	9	Olivier Argagnon
EU-AT-001	Austrian Vegetation Database	8	Wolfgang Willner
EU-NL-001	Dutch National Vegetation Database	8	Stephan Hennekens

EU-RS-003 + EU-RS-004	Database of Forest Vegetation in Republic of Serbia + Vegetation Database of Northern Part of Serbia (AP Vojvodina)	8	Mirjana Krstivojević Ćuk
00-TR-001	Forest Vegetation Database of Turkey - FVDT	8	Ali Kavgacı
EU-RS-002	Vegetation Database Grassland Vegetation of Serbia	7	Svetlana Aćić
EU-SI-001	Vegetation Database of Slovenia	7	Urban Šilc
NA	Bosnia and Herzegovina	7	Vladimir Stupar
AS-TR-002	Vegetation Database of Oak Communities in Turkey	6	Emin Uğurlu
EU-00-016	Mediterranean Ammophiletea database	4	Corrado Marcenò
EU-DE-014	German Vegetation Reference Database (GVRD)	4	Ute Jandt
EU-ME-001	Vegetation Database of Montenegro	4	Milica Stanišić-Vujačić
EU-DE-013	VegetWeb Germany	3	Florian Jansen
EU-GR-006 + EU-GR-007	Hellenic Woodland Database + Hellenic Beech Forests Database (Hell-Beech-DB)	3	Ioannis Tsiripidis
EU-00-002	Nordic-Baltic Grassland Vegetation Database (NBGVD)	3	Jürgen Dengler
EU-PL-001	Polish Vegetation Database	3	Zygmunt Kącki
EU-00-023	Iberian and Macaronesian Vegetation Information System (SIVIM) – Deciduous Forests	3	Juan Antonio Campos
EU-IT-010	Vegetation database of Habitats in the Italian Alps - HabItAlp	2	Laura Casella
NA	Steppe vegetation Rostov Region Database	2	Olga Demina
EU-CH-011	Monitoring Effectiveness of Habitat Conservation in Switzerland	2	Ariel Bergamini
EU-BE-002	INBOVEG	1	Sophie Vermeersch
EU-BG-001	Bulgarian Vegetation Database	1	Iva Apostolova
EU-00-017	European Coastal Vegetation Database	1	John Janssen
EU-DE-020	German Grassland Vegetation Database (GrassVeg.DE)	1	Ricarda Pätsch
EU-00-021	SE Europe forest database	1	Andraž Čarni
EU-00-020	WetVegEurope	1	Flavia Landucci
EU-00-004	Iberian and Macaronesian Vegetation Information System (SIVIM)	1	Borja Jiménez-Alfaro
EU-MK-001	Vegetation Database of the Republic of Macedonia	1	Renata Ćušterevska
