# Supplementary material for: Different Ecological Niches of Poisonous Aristolochia clematitis in Central and Marginal Distribution Ranges—Another Contribution to a Better Understanding of Balkan Endemic Nephropathy
Source: Plants (Basel). 2023 Aug 22;12(17):3022. doi: 10.3390/plants12173022 (PMC10489678; doi:10.3390/plants12173022)
Supplement: Supplementary file 1 [file plants-12-03022-s001.zip › Table S2.pdf]

Table S2. Descriptive statistics for the bioclimatic variables calculated on a whole dataset (851 vegetation plots)

Variable		Arithmetic Mean	SD	Min	Max
BIO1	Mean annual air temperature °C	10.8	2.7	0.8	18.1
BIO2	mean diurnal air temperature range °C	8.3	1.4	2.4	11.4
BIO3	Isothermality °C	0.3	0.0	0.1	0.4
BIO4	Temperature seasonality °C/100	795.5	152.5	470.5	1187.1
BIO5	Mean daily maximum air temperature of the warmest month °C	26.4	2.4	15.5	33.5
BIO6	Mean daily minimum air temperature of the coldest month °C	-3.6	4.1	-14.4	7.2
BIO7	Annual range of air temperature °C	30.0	4.3	16.6	39.8
BIO8	Mean daily mean air temperatures of the wettest quarter °C	15.7	5.5	-0.7	23.2
BIO9	Mean daily mean air temperatures of the driest quarter °C	7.4	9.2	-7.5	26.6
BIO10	Mean daily mean air temperatures of the warmest quarter °C	21.0	2.2	10.0	26.6
BIO11	Mean daily mean air temperatures of the coldest quarter °C	0.6	4.2	-10.9	10.4
BIO12	Annual precipitation amount kg m^{-2} year	714.4	293.3	275.7	2233.7
BIO13	Precipitation amount of the wettest month kg m^{-2} $month^{-1}$	88.5	36.5	29.8	250.2
BIO14	Precipitation amount of the driest month kg m^{-2} $month^{-1}$	36.1	17.4	6.4	122.2
BIO15	Precipitation seasonality kg m^{-2}	26.9	10.8	10.8	85.9
BIO16	Mean monthly precipitation amount of the wettest quarter kg m^{-2} $month^{-1}$	241.6	103.3	82.7	726.7
BIO17	Mean monthly precipitation amount of the driest quarter kg m^{-2} $month^{-1}$	123.4	58.5	25.1	412.6
BIO18	Mean monthly precipitation amount of the warmest quarter kg m^{-2} $month^{-1}$	185.9	71.8	25.1	579.9
BIO19	Mean monthly precipitation amount of the coldest quarter kg m^{-2} $month^{-1}$	164.4	94.9	61.2	560.2
